# Do Children Receive Equal Justice Under the Law? A Comparison of Sentence Severity for Crimes with Child and Adult Victims

**DOI:** 10.1007/s42448-025-00234-2

**Published:** 2025-09-01

**Authors:** Sarah Henry Davis, Sarah A. Font

**Affiliations:** 1 Pennsylvania State University, State College, USA; 2 The Brown School, Washington University, St. Louis, USA

**Keywords:** Sentencing, Criminal justice, Child abuse, Sexual assault

## Abstract

This study examines the differences in sentencing for offenses involving child victims versus adult victims within a statewide population of adults convicted of physical and sexual assault. Crimes against children are often viewed as particularly heinous and may consequently receive harsher sentences. However, children may be unreliable witnesses or unable to testify, leading to more lenient sentencing through plea agreements. This study investigates the disparities in sentencing for physical and sexual violence against children in comparison to adults. We utilize data from the Pennsylvania Commission on Sentencing, which includes 47,288 adults convicted of physical and sexual assaults between 2014 and 2019. The sentencing outcomes encompass sentence type (incarceration versus noncustodial penalty) and severity (in relation to the presumptive sentence under guidelines). Logistic and linear regression models are employed to adjust for offender demographics and case characteristics. The findings indicate that physical assaults against children are punished less severely than those against adults, while sexual assaults against children receive harsher penalties than sexual assaults against adults. The implications for policy and practice are discussed.

**Supplementary Information:**

The online version contains supplementary material available at 10.1007/s42448-025-00234-2.

## Introduction

A substantial portion of children, both worldwide and in the USA specifically, experience some form of physical or sexual abuse before the age of 18 (Brooker et al., 2001; Finkelhor, 1994; Massullo et al., 2023; Norman et al., 2012; Stoltenborgh et al., 2011). In national surveys, one in six people self-reported childhood physical abuse, and one in ten reported childhood sexual abuse (Centers for Disease Control & Prevention, 2019).

Exposure to abuse, whether physical or sexual, has substantial negative impacts on children, including depression, anxiety, PTSD, and various other debilitating physical and mental health conditions (Ehring et al., 2014; Emery & Laumann-Billings, 1998; Golding, 1999; Kendall-Tackett et al., 1993; Radell et al., 2021; Springer et al., 2007). These adverse victim impacts also have spillover effects on society: research suggests a strong link between childhood abuse and substance use, aggression, and criminal behavior (Mathews et al., 2020; Norman et al., 2012).

The criminal justice system imposes sanctions, including incarceration, on offenders who victimize children. Nevertheless, within the criminal justice system, studies consistently show that victim characteristics matter—that the severity of punishment an offender receives varies based on the race, gender, or class of the victim (Curry et al., 2004). Although not widely studied, the victim’s age may also affect how offenders are sentenced. Children are perceived as innocent, and thus, crimes against them may be viewed as especially egregious. Moreover, as not fully autonomous persons, children may be less likely to be blamed for their victimization, especially sexual victimization (Erez & Tontodonato, 1990). At the same time, children may also be perceived as less credible witnesses than adults, leading to more lenient plea offers. In cases of physical abuse, where most offenders are parents, judges may view the abuse as less serious than physical assaults of adults because, unlike parents, they can legally use violence against children; whether such violence is criminal is a question of degree rather than category.

Yet, few studies have examined sentencing for crimes against children or compared sentencing severity for crimes involving children versus adults. This study considers the following question: In physical and sexual assault cases, are offenders with child victims sentenced differently from offenders with adult victims? We specifically consider three dimensions of sentencing: the decision to incarcerate (versus applying other criminal sanctions, such as probation), the length of incarceration, and judges’ deviation from sentencing guidelines.

## Background

Numerous factors influence criminal sentencing. The most explicit legal factors in sentencing are the severity of the offense and the history of the offender. In Pennsylvania, sentencing guidelines reduce unwarranted variation in sentencing—specifically disparities in sentencing of the same offense across racial/ethnic groups. Nevertheless, court actors have a substantial influence on the type and length of sentences. For example, prosecutors can manipulate charges to avoid mandatory sentencing guidelines (Ulmer et al., 2007). Of particular interest in this study, prosecutors make sentencing recommendations following a plea bargain or trial, and judges exercise discretion in the sentence imposed.

Previous research on sentencing variation has primarily focused on the race and gender of the offender, typically finding that male and Black or Hispanic offenders receive harsher sentences than female or White offenders (Ulmer et al., 2020). More recent research considers whether offenders are sentenced more harshly or leniently based on victim characteristics, focused mainly on victim race and gender (Holcomb et al., 2004; Ulmer et al., 2020; Williams et al., 2008). There are many reasons that victim characteristics may influence sentencing decisions. First, victims may vary in their perceived credibility or their capacity to testify clearly. In cases of physical assault and, especially, sexual assault, the victim’s testimony is often the most critical evidence. If the victim’s testimony is questionable, prosecutors are more likely to offer a plea with a lenient sentencing recommendation to avoid risking an acquittal at trial (Lewis et al., 2014). Research findings are discordant with regard to the perceived credibility of child victims, with studies reporting that younger children are perceived as more credible than older children (Voogt et al., 2020) and others highlighting skepticism about the testimony of young children (Newcombe & Bransgrove, 2007). In the 1980 s, as recognition of the prevalence of child sexual abuse rose, there was a simultaneous rise in false cases of sexual abuse, in which children were often inadvertently manipulated into making false disclosures of horrific abuse (Edwards & Lohman, 1994; Victor, 1998). Although many reforms were enacted to avoid false disclosures—such as ensuring children are interviewed by qualified experts who avoid leading questions—these cases left lingering doubt about the credibility of child witnesses.

Second, some victims may evoke more sympathy from prosecutors and judges, resulting in harsher sentences. A study of death penalty decisions found that offenders who killed children were more likely to receive the death penalty than those whose victims were older (Marier et al., 2018), suggesting that child victims may inflame the emotions of decision-makers more so than adult victims. However, historically, children have been viewed as the property of their parents (Woodhouse, 1991). Because parents and other caregivers are the primary perpetrators of physical violence against children, the status of children as property or, at least, dependents—whose treatment is primarily left to the discretion of parents in all but the most egregious cases—may reduce the perceived severity of physical assault cases involving child victims.

Lastly, prosecutors may consider the emotional impact of a trial on the victim when deciding how lenient a plea offer will be made to the offender. Trials can impose substantial emotional hardship on victims of violent crimes due to having to relive their experience in front of the offender and strangers and being subject to aggressive cross-examination by defense attorneys who question their credibility and behavior (Reed & Caraballo, 2022; Steketee & Austin, 1989). Because children are perceived as more vulnerable or likely to be distraught by testifying in the presence of their abuser, prosecutors may make greater efforts—via more generous plea offers—to avoid a trial in cases with child victims.

In sum, whether the victim is a child or an adult can influence the perceived credibility of the case as well as the severity of the offense. In this study, we examine if and how sentencing of physical and sexual violence differs for cases involving child versus adult victims in Pennsylvania. Because there are competing arguments as to whether sentencing would be more or less severe for cases with child victims compared with adult victims, we pose no a priori hypotheses.

## Methods

### Data

The data for this study were from the Pennsylvania Commission on Sentencing (PCS). The dataset included offender, offense, and sentencing information for all adult misdemeanor or felony criminal convictions—except for 1st- and 2nd-degree murder convictions—between 2014 and 2019 in Pennsylvania. The dataset comprised county submissions to a web-based reporting system, aggregated and validated by the PCS, where data entry often involved a mix of drop-down selections for structured variables (such as demographics, prior record score, and offense gravity score) and free-text fields for offense descriptions. Our analysis was all cases involving physical or sexual assault (*N* = 47,288). Details about offense coding are described below.

### Dependent Variables

The dependent variables pertained to sentence type and punitiveness. First, the dependent variable of incarceration was a binary variable coded as 1 if the offender was sentenced to any length of time in jail or prison and 0 if an alternative sentence was chosen (e.g., probation, intermediate sanctions). Second, *length of incarceration* was a continuous variable measured by the number of months an offender was sentenced to jail or prison. Individuals not sentenced to jail or prison (such as those receiving probation or fines) had a value of 0 on this measure. Third, we considered whether a sentence for a particular defendant was more or less punitive than expected, given the offense for which the defendant was convicted and the defendant’s criminal history. The PCS established guidelines to reduce disparities in sentencing among individuals with similar records who committed the same criminal offense. Thus, for each case, there was a minimum presumptive sentence equal to the lowest expected length (in months) of incarceration, given the offense severity and prior criminal record, and a maximum presumptive sentence equal to the upper bound of the sentencing range. For example, for an offense gravity of 10 and a prior record score of 2, the sentencing guideline ranged from 36 months (the minimum presumptive sentence) to 48 months (the maximum presumptive sentence). These variables were equal to 0 if the presumptive sentence was noncustodial. This means that if the sentencing guidelines for a particular offense and criminal history combination recommended a non-incarceration penalty (e.g., probation, fines), then the numerical value for the minimum and maximum presumptive sentence length (in months) was recorded as 0. Using these variables (minimum and maximum presumptive sentences), we created two measures of sentence punitiveness. *Sentence exceeding minimum* was the number of months that the offender was incarcerated above the minimum presumptive sentence. That is, this measure was equal to the difference between the actual sentence and the minimum sentence guideline for everyone sentenced above the minimum and equal to 0 for anyone sentenced at or below the minimum sentence. The *upward departure* measure was binary and equal to 1 if the sentence exceeded the maximum sentence guideline and 0 otherwise.

### Independent Variables

We sought to identify two types of criminal offenses, physical assaults and sexual assaults, and to discern for each offense type whether the victim was an adult or a child. Measures were created by manual coding of the offense description variable in the dataset to identify sexual assaults, physical assaults, and the crimes within these categories that pertained to a child victim. Some specific offense descriptions or charge codes from the database that were categorized as physical assault are “Aggravated Assault,” “Simple Assault,” and “Child Abuse-Physical,” and for sexual assault, “Rape,” “Sexual Assault,” “Statutory Sexual Assault,” and “Indecent Assault.” Regarding child victim identification, we primarily relied on specific charge modifier codes that explicitly denoted a child victim (e.g., “Child Victim,” “Victim Under 13”) and keywords within the free-text offense descriptions that referenced the victim’s age (e.g., “victim age 5,” “minor victim”) or used terms like “child,” “juvenile,” or “infant” in the context of the offense. If the victim’s age or status as a child was not explicitly stated or implied by these indicators, the case was not coded as having a child victim. If the crime type (physical vs. sexual assault) or victim age (child vs. adult) was genuinely ambiguous or could not be reliably determined from the available offense descriptions and codes, those specific cases were excluded from our final analytic sample for that particular categorization. The authors met and agreed on these categorizations. One author manually coded the data, while the other provided a double-check of the coding.

In order to make sure the sentence was based primarily on physical or sexual assault, we limited our sample to cases in which the physical or sexual assault was categorized as the most severe offense in that judicial proceeding. The Pennsylvania Commission on Sentencing (PCS) database included an indicator variable identifying the most serious offense in a judicial proceeding, based on the most severe sanction, offense gravity score, and suggested sentence within that proceeding. To ensure our sample captured all relevant cases where physical or sexual assault was a primary component, we included judicial proceedings where a physical or sexual assault charge was present, even if another related, more serious charge (as determined by the offense gravity score) was the “most serious offense” in that proceeding. For instance, in some cases that included a physical assault involving a child victim, the highest-ranking charge was “child endangerment.” Because child endangerment in such a context is intrinsically linked to the physical or sexual assault, we included these cases to avoid undercounting instances where assault was a central act, even if a broader charge carried a higher severity score. Child endangerment can encompass physical or sexual assault, depending on the specific circumstances and charges. Control variables included the offender’s prior criminal record and the severity of the current offense. The prior record score quantified the offender’s criminal history. The score was a weighted scale that incorporated previous felonies and misdemeanors committed by the offender, ranging from 0 (no prior record) to 7 (serious criminal history). The severity of the current offense was measured by the offense gravity score, which ranged from 1 to 14, with 14 being the most serious. The offense gravity score is influenced by various factors, including the loss experienced by the victim and the victim’s level of vulnerability. It is a statutorily mandated and regularly updated measure used across the state’s criminal justice system to classify the severity of offenses that has been extensively used in studies on sentencing.

Demographic controls were race/ethnicity, gender, and age of the offender. Race/ethnicity had three categories: White, Black, and Other (including Hispanic, Native American, and Asian/Pacific Islander). The sex of the offender was coded as a dummy variable, with values of 0 indicating female and 1 indicating male. The offender’s age was recorded as their age in years at the time of sentencing. County urbanicity was included as a control variable to account for potential geographic variations in sentencing practices and community characteristics that might influence judicial discretion or plea bargaining outcomes. Sentencing practices, prosecutorial norms, and even public sentiment (which can indirectly influence sentencing) can vary significantly between different counties and between urban, suburban, and rural areas. Including these controls helped to isolate the effect of victim type by accounting for these confounding geographical factors. County urbanicity was categorized into four categories using the county continuum: large urban, midsize urban, nonmetro, and rural.

### Analyses

Descriptive statistics and *t*-tests were used to assess bivariate differences in offender and sentence characteristics for four groups of offenses: physical assaults against children, physical assaults against adults, sexual assaults against children, and sexual assaults against adults.

Then, regression was used to estimate whether a child victim (versus an adult victim) was associated with the binary sentencing outcomes, net of offender characteristics, offense gravity, and prior record. Logistic regression was used for binary outcomes (incarceration and upward departure), while linear regression was employed for sentence length and lower guide measures. Our regression models estimated the associations between victim type and sentencing outcomes net of (i.e., controlling for) the specified offender demographics and case characteristics. We replicated the OLS models using Tobit regression, which was appropriate for models with censoring on the dependent variable. The results of the Tobit models were consistent with the OLS models. Thus, we presented the simpler and more parsimonious OLS models.

## Results

Of the offenders who were convicted of assault between 2014 and 2019 in Pennsylvania, the majority of convictions were for physical assaults against adults, with convictions for physical assault against children being particularly rare. A majority of offenders (54.8%) received incarceration sentences. For physical assault cases, the share of offenders sentenced to incarceration was similar for cases with child victims (51.3%) versus adult victims (53.5%). Incarceration was more common in cases of sexual assault and differed for cases with child victims: 65.1% of sexual assault cases with adult victims and 82.1% of sexual assault cases with child victims received carceral sentences (Table 1).

**Table 1:** Descriptive statistics

	**Both PA and SA**		**PA child**		**PA adult**		**SA child**		**SA adult**	
*N*	47,288		1272		38,007		4683		3363	
	Mean/%	SD	Mean/%	SD	Mean/%	SD	Mean/%	SD	Mean/%	SD
**Case outcomes**										
Incarcerated (% yes)	54.78		51.26		53.51		82.11***		65.06	
Incarceration length (months)	20.87	34.67	13.83***	18.66	19.19	33.01	44.29***	54.89	29.49	35.42
Maximum presumptive sentence	20.06	38.29	13.72	15.74	18.69	37.91	61.92	82.72	26.07	32.19
Upward departure (% yes)	8.16		7.44		7.99		9.05		10.62	
Minimum presumptive sentence	11.14	24.63	6.28	13.05	10.07	23.31	31.21	44.49	17.57	29.05
Sentenced at minimum (% yes)	36.04		39.03		38.03		9.12		29.57	
Length above the minimum presumptive sentence	2.80	9.50	4.84	7.80	4.98	12.74	11.51***	23.81	6.39	10.89
**Case and offender characteristics**										
Offense gravity score	3.41	2.48	5.17***	1.97***	4.64	3.02	8.46***	3.41	6.33	3.42
Prior record score	1.68	2.04	1.20***	1.79***	1.56	2.01	1.02***	1.79	1.43	2.02
Offender characteristics										
Sex										
Female	13.87		24.16		14.78		5.33		8.18	
Male	86.13		75.84***		85.22		94.67***		91.82	
Age (years)	34.77	11.49	32.57***	9.77	34.40	11.88	36.89	14.42	37.10	13.43
15–24	25.08		23.51		25.23		25.87		20.62	
25–31	25.94		33.49		25.74		20.47		23.70	
32–41	24.48		27.75		24.60		22.14		24.52	
42–99	24.51		15.25		24.42		31.51		31.60	
Race										
White	64.57		64.94		65.86		77.77***		67.50	
Black	33.99		33.65		32.78		20.14***		30.51	
Other	1.43		1.42		1.36		2.09		1.99	
County urbanicity										
Large urban	46.45		45.13		44.81		39.87		44.34	
Midsize urban	30.67		33.88		31.00		30.26		32.05	
Nonmetro	17.23		15.57		18.08		20.59		17.57	
Rural	5.65		5.42		6.11		9.29		6.04	

^*^p < 0.05, **p < 0.01, ***p < 0.0

*CI* for confidence interval, *LL* for lower limit, *UL* for upper limit, *PA* for physical assault, and *SA* for sexual assault

The mean length of incarceration across all assault cases was 20.9 months, but the length varied substantially by case type. Specifically, the mean length for physical assault cases with a child victim was 13.8 months, compared with 19.2 months for physical assault cases with an adult victim. The mean incarceration length for sexual assault cases was higher overall, at 44.3 months for cases with child victims and 29.5 months for cases with adult victims. Due to the skew from outliers, we also examined the median length of incarceration. The overall median length was 9 months. The median months for physical assault of a child was also 9 months, while assault of an adult was 8 months. Looking at sexual assault, the median incarceration length of a child was 18 months, while it was 12 months for an adult victim.

For physical assaults, both the minimum and maximum presumptive sentences were shorter for cases involving child victims than cases with adult victims. In contrast, for sexual assault cases, the minimum and maximum presumptive sentences were longer for sexual assaults with child victims. In addition, the sentence length exceeding the minimum guideline and the proportion of cases with upward departures were statistically equivalent for physical assault cases with child and adult victims. Yet, sexual assault cases with child victims were sentenced, on average, to 11.5 months longer than the minimum presumptive sentence, compared with 6.4 months for sexual assault cases with adult victims. Lastly, for both physical and sexual assaults, cases with child victims had higher offense gravity scores and lower prior record scores than cases with adult victims. Looking at the offenders, the majority were male. However, the percentage of male offenders who committed physical assault against a child was lower than the overall percentage. The race of the offender was generally consistent across crime type and victim age, except for the sexual assault of a child. The percentage of white offenders was significantly higher, and the percentage of black offenders was significantly lower in child sexual assault cases. The age of offenders who committed physical assault against children was also significantly lower.

Table 2 presents the results of logistic regression models predicting sentencing to incarceration for physical and sexual assault cases controlling for all covariates. For physical assault cases, child (versus adult) victim was associated with 27.5% lower odds of incarceration. In contrast, child victim was associated with 15.2% higher odds of incarceration in sexual assault cases. As expected, both offense gravity score and prior record were associated with higher odds of incarceration sentences for both physical and sexual assault cases. Male offenders, compared with female offenders, had 136% higher odds of a sentence to incarceration in sexual assault cases and 56% higher odds in physical assault cases. Younger age at conviction was associated with higher odds of sentencing to incarceration for physical assault cases, but age was not significantly associated with sentencing in sexual assault cases. There was also a gradient by county urbanicity, such that the odds of incarceration were higher for more rural counties.

**Table 2:** Logistic regression of likelihood of being incarcerated for assault

	Physical assault cases(*n* = 39,253)			Sexual assault cases(*n* = 8035)		
	Odds ratio	95% C		Odds ratio	95% CI	
		LL	UL		LL	UL
Child victim (reference: adult victim)	0.725***	[0.639,	0.821]	1.152*	[1.006,	1.318]
Offense gravity score	1.595***	[1.574,	1.615]	1.700***	[1.642,	1.760]
Prior record score	1.372***	[1.353,	1.390]	1.364***	[1.310,	1.420]
Male (reference: female)	1.556***	[1.456,	1.662]	2.366***	[1.904,	2.942]
Offender years of age (reference: 15–24)						
25–31	0.913**	[0.855,	0.975]	1.054	[0.881,	1.261]
32–41	0.859***	[0.802,	0.919]	1.032	[0.860,	1.239]
42–99	0.762***	[0.711,	0.817]	0.922	[0.778,	1.903]
Offender race (reference: White)						
Black	0.994	[0.943,	1.049]	0.982	[0.841,	1.147]
Other	1.292*	[1.038,	1.609]	1.512	[0.933,	2.450]
County urbanicity (reference: large metro)						
Midsize metro	1.651***	[1.562,	1.746]	2.071***	[1.791,	2.395]
Nonmetro	2.080***	[1.947,	2.223]	2.446***	[2.054,	2.914]
Rural	3.342***	[3.016,	3.703]	4.597***	[3.479,	6.075]
Constant	0.055	[0.050,	0.060]	0.021	[0.015,	0.030]

^*^p < 0.05, **p < 0.01, ***p < 0.0

*CI* for confidence interval, *LL* for lower limit, *UL* for upper limit

Table 3 presents the ordinary least squares regression results for length of incarceration and length beyond the minimum sentence guideline, by case type controlling for the full set of covariates described in the methods section (i.e., offender’s prior criminal record, offense gravity score, race/ethnicity, gender, age, and urbanicity). For physical assault cases, child victim was associated with a 5.6-month shorter incarceration length overall and 0.8 fewer months above the minimum guideline. For sexual assault cases, there were no significant differences in overall sentence length by child versus adult victim, but child victim was associated with an incarceration sentence 1.3 months above the minimum guideline. Male (versus female) offenders received longer sentences for physical assault cases only, whereas older offenders and offenders outside of the large metro counties received longer sentences in both case types. Black offenders received significantly shorter incarceration lengths compared to White offenders.

**Table 3:** OLS regression of assault sentence total length and length that exceeds the lower guide level

	Physical assault cases(*n* = 39,251)						Sexual assault cases(*n* = 8031)					
	Total length			Length above minimum presumptive sentence			Total length			Length above minimum presumptive sentence		
	Coefficient	95% CI		Coefficient	95% CI		Coefficient	95% CI		Coefficient	95% CI	
		LL	UL		LL	UL		LL	UL		LL	UL
Child victim (reference: adult victim)	− 5.605***	[− 6.616,	− 4.593]	− 0.796**	[− 1.290,	− 0.301]	− 0.448	[− 1.902,	1.006]	1.345***	[0.560,	2.130]
Offense gravity score	5.986***	[5.926,	6.047]	1.178***	[1.148,	1.208]	9.103***	[8.903,	9.303]	1.782***	[1.674,	1.890]
Prior record score	1.669***	[1.574,	1.764]	− 0.174***	[− 0.221,	− 0.128]	4.612***	[4.237,	4.987]	− 0.562***	[− 0.765,	− 0.360]
Male (reference: female)	1.580***	[1.069,	2.090]	1.047***	[0.797,	1.297]	1.378	[− 1.425,	4.180]	1.377	[− 0.136,	2.891]
Offender years of age (range 15–24)												
25–31	1.009***	[0.504,	1.515]	0.555***	[0.308,	0.803]	4.170***	[2.149,	6.190]	2.059***	[0.968,	3.150]
32–41	1.509***	[0.985,	2.033]	0.737***	[0.481,	0.993]	6.568***	[4.555,	8.581]	3.195***	[2.108,	4.282]
42–99	2.156***	[1.626,	2.685]	0.918***	[0.659,	1.177]	10.232***	[8.346,	12.119]	4.290***	[3.272,	5.309]
Offender race (reference: White)												
Black	− 0.655**	[− 1.063,	− 0.247]	− 0.187239	[− 0.387,	0.012]	− 1.449	[− 3.174,	0.276]	− 0.135	[− 1.067,	0.797]
Other	1.107	[− 0.443,	2.657]	1.183**	[0.425,	1.942]	3.767	[− 1.012,	8.546]	2.958*	[0.378,	5.539]
County urbanicity (reference: large metro)												
Midsize metro	3.253***	[2.827,	3.678]	0.920***	[0.711,	1.128]	3.615***	[1.968,	5.263]	1.755***	[0.865,	2.644]
Nonmetro	3.159***	[2.646,	3.672]	0.780***	[0.529,	1.031]	5.131***	[3.197,	7.065]	1.120*	[0.076,	2.165]
Rural	4.254***	[3.467,	5.041]	1.224***	[0.839,	1.609]	3.894**	[1.197	6.591]	1.009	[− 0.447,	2.466]
Constant	− 24.268	[− 24.947,	− 23.588]	− 4.418	[− 4.751,	− 4.085]	− 54.023	[− 57.410,	− 50.636]	− 11.030	[− 12.859,	−9.201]

^*^p < 0.05, **p < 0.01, ***p < 0.0

*CI* for confidence interval, *LL* for lower limit, *UL* for upper limit

Table 4 presents the logistic regressions predicting upward departures controlling for the full set of covariates. There were no differences in upward departures for child versus adult victims in physical assault cases. In sexual assault cases, a child victim (versus adult) decreased the odds of an upward departure by 16%.

**Table 4:** Logistic regression predicting upward departures in sentencing for assault cases

	Physical assault cases(*n* = 39,251)			Sexual assault cases(*n* = 8031)		
	Odds ratio	95% CI		Odds ratio	95% CI	
		LL	UL		LL	UL
Child victim (reference: adult victim)	0.896	[0.724,	1.109]	0.840*	[0.717,	0.983]
Offense gravity score	1.033***	[1.021,	1.045]	1.009	[0.988,	1.031]
Prior record score	0.735***	[0.716,	0.755]	0.945**	[0.906,	0.986]
Offender sex						
Male (reference: female)	1.630***	[1.453,	1.828]	1.511*	[1.061,	2.151]
Offender years of age (range 15–24)						
25–31	0.869**	[0.787,	0.959]	1.023	[0.808,	1.294]
32–41	0.884*	[0.796,	0.982]	1.318*	[1.054,	1.648]
42–99	0.809***	[0.727,	0.901]	1.338**	[1.084,	1.651]
Offender race (reference: White)						
Black	1.216***	[1.118,	1.322]	1.162	[0.968,	1.395]
Other	1.393*	[1.054,	1.842]	2.375***	[1.606,	3.511]
County urbanicity (reference: large metro)						
Midsize metro	0.970	[0.888,	1.059]	0.906	[0.761,	1.079]
Nonmetro	0.868*	[0.777,	0.969]	0.653***	[0.520,	0.820]
Rural	1.054	[0.900,	1.234]	0.593**	[0.421,	0.835]
Constant	0.074	[0.064,	0.086]	0.074	[0.049,	0.111]

^*^p < 0.05, **p < 0.01, ***p < 0.0

*CI* for confidence interval, *LL* for lower limit, *UL* for upper limit

A higher offense gravity score was associated with a small increase in the odds of upward departure, whereas prior record score was negatively associated with upward departures for both physical and sexual assault cases. The odds of upward departure in physical assault cases were higher for offenders who were male (versus female), 15–24 years old (versus all other age categories), Black (versus White), or in nonmetro (versus large metro) counties. The odds of upward departure in sexual assault cases were higher for offenders who were male (versus female) ages 32 and older (versus 15–24), other race (versus White), or in nonmetro and rural (versus large metro) counties.

### Discussion

This study examined differences in sentencing of physical and sexual assault cases based on whether the victim was a child or adult. Although the sentencing literature has examined numerous victim and offender characteristics as predictors of sentencing, limited attention has been paid to child and adult victims. This was of particular interest given the challenges in prosecuting cases with child victims, as well as the complex history of children’s rights in the USA. Specifically, whereas children have been historically marginalized as parental property, they are also seen as uniquely vulnerable such that offenses against them may be seen as particularly egregious.

Our findings reveal a concerning pattern of sentencing disparity based on victim age, suggesting that the application of justice in Pennsylvania is not entirely uniform. Although sentencing guidelines aim to reduce unwarranted variation, our results suggest that whether the victim is a child or an adult subtly but significantly influences sentencing outcomes. For physical assaults, cases with child victims receive demonstrably shorter incarceration lengths and lower odds of incarceration, implying a more lenient approach. Conversely, sexual assaults against children lead to harsher carceral outcomes, indicating a more punitive stance. This differential treatment, perhaps stemming from societal perceptions of vulnerability or culpability, raises fundamental questions about the equitable application of the law.

The law, in principle, should apply equally regardless of victim characteristics. However, our findings suggest that unwritten sentiments and societal biases may permeate the sentencing process. For instance, the implicit tolerance for certain forms of physical discipline against children, or the intense public outrage surrounding child sexual abuse, may translate into divergent legal outcomes. This highlights a tension between the letter of the law and its practical implementation, where the “just” outcome may be influenced by factors external to the prescribed legal framework.

We found that sentencing disparities for cases with child versus adult victims depended on whether the assault was physical or sexual. Overall, physical assault cases with child victims were less likely to receive incarceration sentences and were sentenced to a shorter length of incarceration. This may reflect that the infliction of physical force against a child must be more severe to be considered an assault, due to policy carveouts for parental (and sometimes school personnel) use of corporal punishment against children (Gershoff & Bitensky, 2007; Gershoff & Font, 2016). Perceptions of parental discipline could create a “zone of acceptable violence” against children that does not exist for adults. Such acceptance of violence against children is consistent with our finding that offenders with child victims received around 5.6 months shorter sentences than offenders with adult victims. Thus, injuries inflicted on children, particularly by parents, must be seen as *excessive* in their use of force to warrant punishment. In contrast, injuries inflicted on adults are likely to be seen as wrong and punishable regardless of the extent of injury. Moreover, physical assaults against children are most often perpetrated by parents, who tend to be sentenced more leniently than non-parents (Hanrath & Font, 2020). Lastly, children who are disabled are far more likely to be the victims of physical abuse, and research suggests that the public is more tolerant of violence against people with disabilities and supports shorter sentencing for harms to children with disabilities specifically (Bottoms et al., 2011; Radkiewicz & Korzeniowski, 2017).

In contrast, sexual assault cases with child (versus adult) victims were more likely to receive incarceration sentences and to be sentenced above the minimum, though less likely to receive upward departures. On the whole, findings of harsher sentencing for sexual offenses against children were consistent with heightened public fear of child sex offenders and support for policies that restrict their liberties (Kernsmith et al., 2009). An increased fear leads to a greater support of harsher sentencing practices in order to mitigate outrage. This also translates to how court actors respond to child sexual assault cases. Judges can be influenced by public perception in determining sentencing, and may be especially reluctant to be seen as “soft on crime” in the context of child sex offenses (Pickett, 2019). This can be especially true in states like Pennsylvania, where judges are chosen electorally. This support from the general public and court actors, such as judges, is consistent with our findings that sexual assault offenders with child victims were 15.2% more likely to receive incarceration than those with adult victims (Table 2) and were sentenced 1.3 months longer than the minimum guideline (Table 3). However, we note that there does appear to be a paradox in that, despite longer sentences for sex offenders with child victims, these offenders are less likely to receive an upward departure. Perhaps, the initial guidelines for child sexual assault are already quite high, limiting the perceived need for or utility of upward departures.

Beyond victim age, our findings also illuminate persistent patterns of sentencing disparity related to offender characteristics, particularly sex and race. Consistent with prior research, male offenders faced significantly higher odds of incarceration across both physical (56% higher odds) and sexual assault cases (136% higher odds) compared to female offenders (Table 2). This aligns with established patterns of gender-based sentencing, where men typically receive harsher penalties, especially for violent crimes. However, the magnitude of this disparity was notably larger in sexual assault cases, suggesting that the perceived severity of this crime, combined with societal expectations of male perpetrators, may lead to particularly punitive outcomes for men. 

The role of offender race presented a more complex picture. Although Black offenders in physical assault cases had 1.216 times higher odds of upward departure (Table 4) compared to White offenders, they also received significantly shorter overall incarceration lengths (Table 3). This seemingly contradictory finding warrants further exploration. It is possible that plea bargaining dynamics or the nature of the specific physical assault cases varies by race in ways unobserved in this study. Such omitted variables may produce ambiguous or paradoxical findings. Conversely, in sexual assault cases, racial disparities were less pronounced for incarceration likelihood and length, but “Other” race offenders had 2.375 times higher odds of upward departure (Table 4) compared to White offenders, indicating that racial and ethnic biases persist, albeit in varied forms depending on offense type.

The finding that physical assaults against children are punished less severely, coupled with the observation that Black offenders receive shorter incarceration lengths for physical assault, suggests a complex interplay of factors that may compound or mitigate disparities. Future research in other states or regions should determine whether these Pennsylvania patterns regarding victim age and other factors are found elsewhere. In addition, studies with other criminal case data—including charges that are ultimately dismissed—may enable researchers to fully disentangle how offender demographics, offense type, and victim age collectively shape criminal case processing. In addition, qualitative research, such as interviews with judges, prosecutors, defense attorneys, or prospective jurors, may help to understand the decision-making processes and underlying rationales that contribute to disparate case outcomes.

### Limitations

We note several limitations. First, the analysis was limited to a single state, Pennsylvania, and states differ in the level of discretion given to judges in sentencing and other characteristics of the legal system. Second, we lacked information on the strength of the evidence or more in-depth knowledge of how plea agreements were reached (most cases in this study were resolved by plea rather than trial). Relatively few child physical and sexual abuse cases result in criminal prosecution, much less conviction (Block, 2019; Hanrath & Font, 2020). Consequently, our sample of convictions was relatively selective and likely to reflect the most egregious cases or cases with unusually strong evidence, such as a confession or an eye witness. Third, we lacked information about the victim-offender relationship, which was likely to influence case processing. Fourth, our analysis of offender race/ethnicity was limited by the categorical nature of the data available in the PCS database, which broadly grouped individuals into “White,” “Black,” and “Other” (including Hispanic, Native American, and Asian/Pacific Islander). This lack of adequate striation of participants’ ethnicity prevents a more nuanced examination of how specific ethnic groups might experience differential sentencing outcomes, particularly given the known complexities of racial and ethnic disparities within the criminal justice system. Future research should strive to utilize datasets with more granular demographic information to explore these critical intersections more thoroughly. Lastly, the charge modifier code that allowed us to identify physical assaults against children may have been underutilized or used differently across jurisdictions. If so, we may have under-identified the share of physical assault cases with child victims.

### Conclusion and Policy Implications

Children are highly vulnerable to victimization by adults, but the legal system faces numerous challenges in prosecuting individuals who harm children. Many of the best practices for handling child abuse cases, such as timely forensic interviews and medical exams at a Child Advocacy Center, are not used consistently due to lack of availability. If sentencing disparities for child victims reflect more lenient plea agreements, improving the capacity of law enforcement and other entities to investigate child abuse cases may help increase the strength of evidence available to prosecutors. Strengthening victim support services, especially for child victims, could reduce reliance on plea bargains driven by concerns about child testimony, potentially leading to more consistent sentencing. A potential policy improvement could be training for judges and prosecutors on unconscious biases related to victim age and child physical assault cases involving familial perpetrators. In addition, public attitudes in the USA continue to be relatively permissive about the use of violence to discipline children, which creates challenges in preventing and responding to physical abuse of children. Public education campaigns may help to shift attitudes regarding corporal punishment and violence against children, which may indirectly influence legal outcomes. Enhanced efforts to educate the public on the nature and life-long ramifications of physical abuse may be needed. Despite efforts to reduce the amount of corporal punishment in school and daycare settings, more could be done to change attitudes and practices surrounding punishment of children.

In conclusion, our study provides compelling evidence that children do not receive equal justice under the law in Pennsylvania following physical and sexual assault victimization. The observed disparities, where physical assaults against children are punished less severely and sexual assaults against children receive harsher penalties, underscore a system where victim characteristics, particularly age, play a significant but inconsistent role. This raises fundamental questions about equal protection under the law for both victims and offenders, as sanctions reflect factors other than the severity of the crime or the offender’s record. Addressing this requires a multi-faceted approach, including a critical examination of existing sentencing guidelines, enhanced training for legal professionals to mitigate implicit biases, and broader public education campaigns to challenge societal norms that may inadvertently contribute to these disparities. Ultimately, ensuring true equity in the criminal justice system necessitates a commitment to apply the law consistently, regardless of who the victim is.

## Supplementary Information

Below is the link to the electronic supplementary material.

MOESM 1 (DOCX 29 KB)

## Data Availability

Data in this manuscript comes from the Pennsylvania Commission on Sentencing. Data sets from the Commission, either General or Contracted Release, must be submitted in writing using the Data Set:  Sentencing Data Request Form, available on the Commission’s website (https://pasentencing.us).
