# Supplementary material for: Do Children Receive Equal Justice Under the Law? A Comparison of Sentence Severity for Crimes with Child and Adult Victims
Source: Int J Child Maltreat. 2025 Sep 1;8(4):437–54. doi: 10.1007/s42448-025-00234-2 (PMC12644201; doi:10.1007/s42448-025-00234-2)
Supplement: Supplementary file 1 — (DOCX 29 KB) [file 42448_2025_234_MOESM1_ESM.docx]

**Appendix**

Supplementary analyses of Physical assault matching Offense Gravity Score

| **A1.Logistic Regression of Likelihood of being Incarcerated for Physical Assault (by OGS)** | | | |  |
| --- | --- | --- | --- | --- |
|  |  |  |  |  |
|  | Odds ratio | 95% CI | |  |
|  |  | LL | UL |  |
| Child Victim | 0.605*** | [0.510, | 0.718] |  |
| OGS | 2.004*** | [1.858, | 2.162] |  |
| PRS | 1.381*** | [1.327, | 1.437] |  |
| Male | 1.617*** | [1.362, | 1.920] |  |
|  |  |  |  |  |
| Offender Age |  |  |  |  |
| 25-31 | 0.941 | [0.788, | 1.123] |  |
| 32-41 | 0.907 | [0.753, | 1.092] |  |
| 42-99 | 0.837 | [0.679, | 1.005] |  |
|  |  |  |  |  |
| Offender Race |  |  |  |  |
| black | 1.059 | [0.913, | 1.228] |  |
| other | 1.475 | [0.869, | 2.502] |  |
|  |  |  |  |  |
| County |  |  |  |  |
| Urban | 2.285*** | [1.964, | 2.568] |  |
| Rural | 3.168*** | [2.617, | 3.834] |  |
| More Rural | 5.031*** | [3.710, | 6.824] |  |
|  |  |  |  |  |
| _cons | 0.016 | [0.010, | 0.028] |  |

| **A2.OLS Regression of Assault Sentence Total Length and Length that Exceeds the Lower Guide Level (by OGS)** | | | | | | |  |
| --- | --- | --- | --- | --- | --- | --- | --- |
|  |  |  |  |  |  |  |  |
|  |  | Physical |  |  | Sexual |  |  |
|  | Coefficient | 95% CI | | Coefficient | 95% CI | |  |
|  |  | LL | UL |  | LL | UL |  |
| Child Victim | -1.233* | [-2.322, | -0.146] | -0.568* | [-1.076, | -0.061] |  |
| OGS | 2.689*** | [2.234, | 3.143] | 0.532*** | [0.320, | 0.744] |  |
| PRS | 2.310*** | [2.112, | 2.507] | -0.214*** | [-0.307, | -0.122] |  |
| Male | 2.158*** | [1.050, | 3.266] | 1.591*** | [1.074, | 2.109] |  |
|  |  |  |  |  |  |  |  |
| Offender Age |  |  |  |  |  |  |  |
| 25-31 | 1.204* | [0.148, | 2.259] | 0.661** | [0.169, | 1.154] |  |
| 32-41 | 0.627 | [-0.478, | 1.732] | 0.771** | [0.256, | 1.287] |  |
| 42-99 | 1.976*** | [0.876, | 3.077] | 1.082*** | [0.569, | 1.596] |  |
|  |  |  |  |  |  |  |  |
| Offender Race |  |  |  |  |  |  |  |
| black | -0.327 | [-1.210, | 0.555] | 0.142 | [-0.270, | 0.553] |  |
| other | 0.508 | [-2.453, | 3.469] | 0.979 | [-0.403, | 2.360] |  |
|  |  |  |  |  |  |  |  |
| County |  |  |  |  |  |  |  |
| Urban | 1.860*** | [0.953, | 2.766] | 0.461* | [0.038, | 0.884] |  |
| Rural | 2.503*** | [1.437, | 3.569] | 0.610* | [0.112, | 1.107] |  |
| More Rural | 4.481*** | [2.968, | 5.993] | 1.080** | [0.374, | 1.785] |  |
|  |  |  |  |  |  |  |  |
| _cons | -13.880 | [-17.008, | -10.752] | -1.955 | [-3.415, | -0.496] |  |

| **A3.Logistic Regression of Assault Sentences that Exceed the Upper Guide Level (by OGS)** | | | |  |
| --- | --- | --- | --- | --- |
|  |  |  |  |  |
|  | Odds ratio | 95% CI | |  |
|  |  | LL | UL |  |
| Child Victim | 1.105 | [0.830, | 1.471] |  |
| OGS | 1.056 | [0.938, | 1.189] |  |
| PRS | 0.976 | [0.926 | 1.027] |  |
| Male | 2.549*** | [1.705, | 3.811] |  |
|  |  |  |  |  |
| Offender Age |  |  |  |  |
| 25-31 | 1.076 | [0.806, | 1.435] |  |
| 32-41 | 1.224 | [0.914, | 1.640] |  |
| 42-99 | 1.267 | [0.950, | 1.689] |  |
|  |  |  |  |  |
| Offender Race |  |  |  |  |
| black | 0.941 | [0.742, | 1.194] |  |
| other | 2.419** | [1.392, | 4.204] |  |
|  |  |  |  |  |
| County |  |  |  |  |
| Urban | 0.930 | [0.734, | 1.180] |  |
| Rural | 0.898 | [0.675, | 1.194] |  |
| More Rural | 0.968 | [0.652, | 1.435] |  |
|  |  |  |  |  |
| _cons | 0.023 | [0.009, | 0.054] |  |
